# Segmental strain analysis for the detection of chronic ischemic scars in non-contrast cardiac MRI cine images

**DOI:** 10.1038/s41598-021-90283-7

**Published:** 2021-06-11

**Authors:** M. Polacin, M. Karolyi, M. Eberhard, A. Gotschy, B. Baessler, H. Alkadhi, S. Kozerke, R. Manka

**Affiliations:** 1 Institute of Diagnostic and Interventional Radiology, University Hospital Zurich, University of Zurich, Raemistrasse 100, 8091 Zurich, Switzerland; 2 Department of Cardiology, University Heart Center, University Hospital Zurich, University of Zurich, Raemistrasse 100, 8091 Zurich, Switzerland; 3 Institute for Biomedical Engineering, University and ETH Zurich, Gloriastrasse 35, 8092 Zurich, Switzerland

**Keywords:** Cardiology, Medical research

## Abstract

Cardiac magnetic resonance imaging (MRI) with late gadolinium enhancement (LGE) is considered the gold standard for scar detection after myocardial infarction. In times of increasing skepticism about gadolinium depositions in brain tissue and contraindications of gadolinium administration in some patient groups, tissue strain-based techniques for detecting ischemic scars should be further developed as part of clinical protocols. Therefore, the objective of the present work was to investigate whether segmental strain is noticeably affected in chronic infarcts and thus can be potentially used for infarct detection based on routinely acquired non-contrast cine images in patients with known coronary artery disease (CAD). Forty-six patients with known CAD and chronic scars in LGE images (5 female, mean age 52 ± 19 years) and 24 gender- and age-matched controls with normal cardiac MRI (2 female, mean age 47 ± 13 years) were retrospectively enrolled. Global (global peak circumferential [GPCS], global peak longitudinal [GPLS], global peak radial strain [GPRS]) and segmental (segmental peak circumferential [SPCS], segmental peak longitudinal [SPLS], segmental peak radial strain [SPRS]) strain parameters were calculated from standard non-contrast balanced SSFP cine sequences using commercially available software (Segment CMR, Medviso, Sweden). Visual wall motion assessment of short axis cine images as well as segmental circumferential strain calculations (endo-/epicardially contoured short axis cine and resulting polar plot strain map) of every patient and control were presented in random order to two independent blinded readers, which should localize potentially infarcted segments in those datasets blinded to LGE images and patient information. Global strain values were impaired in patients compared to controls (GPCS p = 0.02; GPLS p = 0.04; GPRS p = 0.01). Patients with preserved ejection fraction showed also impeded GPCS compared to healthy individuals (p = 0.04). In patients, mean SPCS was significantly impaired in subendocardially (−  5.4% ± 2) and in transmurally infarcted segments (− 1.2% ± 3) compared to remote myocardium (− 12.9% ± 3, p = 0.02 and 0.03, respectively). ROC analysis revealed an optimal cut-off value for SPCS for discriminating infarcted from remote myocardium of − 7.2% with a sensitivity of 89.4% and specificity of 85.7%. Mean SPRS was impeded in transmurally infarcted segments (15.9% ± 6) compared to SPRS of remote myocardium (31.4% ± 5; p = 0.02). The optimal cut-off value for SPRS for discriminating scar tissue from remote myocardium was 16.6% with a sensitivity of 83.3% and specificity of 76.5%. 80.3% of all in LGE infarcted segments (118/147) were correctly localized in segmental circumferential strain calculations based on non-contrast cine images compared to 53.7% (79/147) of infarcted segments detected by visual wall motion assessment (p > 0.01). Global strain parameters are impaired in patients with chronic infarcts compared to controls. Mean SPCS and SPRS in scar tissue is impeded compared to remote myocardium in infarcts patients. Blinded to LGE images, two readers correctly localized 80% of infarcted segments in segmental circumferential strain calculations based on non-contrast cine images, in contrast to only 54% of infarcted segments detected due to wall motion abnormalities in visual wall motion assessment. Analysis of segmental circumferential strain shows a promising method for detection of chronic scars in routinely acquired, non-contrast cine images for patients who cannot receive or decline gadolinium.

## Introduction

Myocardial infarction (MI) often results in irreversible scar formation of the myocardium. Cardiac magnetic resonance imaging (MRI) with late gadolinium enhancement (LGE) is considered the gold standard method for detection and visualization of scar tissue after MI^1,2^. To this end, intravenous application of gadolinium-based contrast agents is required for visualizing scar tissue, as there are currently no alternatives in cardiac MRI for this task. LGE sequences are time consuming and typically use up more than 50% of the exam time due to the required 10–15 min time delay after contrast agent administration, which is important for contrast retention in scar tissue^3^. Moreover, the intravenous application of gadolinium-based contrast agents is restricted in patients with acute and chronic renal failure^4^. Additionally, intravenous contrast agent application may cause an allergic reaction in some circumstances, which can be life threatening^5^. Finally, recent studies suggest possible deposition of linear gadolinium chelates, e.g. in brain and bone^6,7^, which is nurturing uncertainty among both patients and treating physicians. Therefore, alternative scar detection methods based on routinely acquired cine images increasingly gain attention^8–10^.

During cardiac contraction, myocardial deformation can be described by vectors in the radial, circumferential and longitudinal directions. In healthy myocardium, negative strain values are measured for circumferential and longitudinal direction during systole, while radial strain yields positive values due to thickening in the radial direction during ventricular contraction^11^. Scar tissue leads to regionally altered strain behavior of the myocardium due to reduced contractility of myofibroblasts, which replace myocytes after infarction^12^.

Different techniques for measuring global and regional myocardial deformation have been developed in the past two decades, like myocardial tagging^13,14^, tissue displacement encoding with stimulated echoes^15^ and strain encoded imaging^16^. All these techniques—with myocardial tagging being the reference modality for evaluating myocardial strain—have in common, that sequences need to be acquired additionally to an already long clinical protocol. Myocardial feature tracking (FT) was introduced for myocardial strain quantification using routinely acquired steady-state free precession (SSFP) cine sequences as input^9,17–19^. Based on optical flow methods^20^ or non-rigid algorithm for image registration and segmentation^21,22^, myocardial borders can be identified and displacement of myocardial segments can be tracked throughout the cardiac cycle.

Recent studies focused on the investigation of global strain parameters in patients with acute and chronic infarcts, revealing impaired global longitudinal and global circumferential strain in these patients^23,24^. Studies that analyzed segmental strain in patients with ischemic scars show heterogenous results^25–27^, in particular reduced accuracy and reproducibility of segmental strain was reported. Those studies typically used optical flow- based FT methods, where only previously defined myocardial boundaries are tracked. The FT software used in this study is based on an elastic algorithm for image registration with tracking of the entire image content with higher reliability, accuracy and interobserver agreement for segmental strain^22,28^. The purpose of this study was to examine global and segmental strain in patients with chronic infarcts and healthy controls and to investigate whether chronic scar tissue affects segmental strain and thus can potentially be used for detection of chronic infarcts based on routinely acquired non-contrast cine images in patients with known coronary artery disease (CAD).

## Methods

### Study population

From September 2018 to June 2019 46 patients (5 female, mean age 52 ± 19 years) with known CAD (diagnosis of CAD since > 7 month), ischemic scars (> 4 months old) visible in standard LGE images, fully diagnostic datasets and signed informed consent were used for this retrospective study. Patients with concomitant primary cardiomyopathies (n = 4) were not enrolled, since strain values might be altered in those patients^11^. Furthermore, patients with unstable angina or signs of acute cardiac insufficiency (n = 29), reduced image quality (n = 34) and patients without signed informed consent (n = 198) were not enrolled. A control group of 24 age- and gender matched individuals (2 female, mean age 47 ± 13 years) with normal cardiac MRI findings were also retrospectively enrolled during the same time period. Causes for referral in the control group were exclusion of structural heart disease (n = 2) or exclusion of coronary artery disease (n = 22). This study was conducted in accordance to the Declaration of Helsinki and its later amendments and the institutional review board approved this retrospective study (Cantonal ethics commission Zurich, BASEC-Nr. 2019-00808). Data including image material were handled anonymously.

### CMR data acquisition

CMR was performed on a 1.5 T MR system (Achieva, Philips Healthcare, Best, the Netherlands) using a dedicated 5-channel phased array coil. Cine balanced SSFP pre-contrast images in standard long-axis geometries (two-, three- and four-chamber view) as well as in short-axis orientation covering the entire left ventricle (LV) were acquired (field of view: 350 × 350 mm^2^, matrix: 300 × 300, repetition time/echo time: 3.0/1.5 ms, in-plane resolution, 1.5 × 1.5 mm^2^; number of cardiac phases: 25–50, section thickness: 8 mm). LGE images (inversion recovery gradient-echo sequence: field of view: 350 × 350 mm^2^; matrix: 256 × 256; repetition time/echo time: 7.4/4.4; inversion time: 205–250 ms; flip angle: 20°; in-plane resolution: 1.5 × 1.5 mm^2^; section thickness: 8 mm) covering the entire LV in short axis view as well as in 2-, 3- and 4 chamber view were acquired 15 min after administration of a bolus of 0.2 mmol of gadobutrol (Gadovist; Bayer Schering Pharma, Zurich, Switzerland) per kilogram body weight.

### CMR data analysis

#### Assessment of infarcted segments in LGE images

After calculation of ventricular volumes and function (IntelliSpace Portal, Philips, Version 8.0.3) (Table 1), infarcted segments were identified on LGE images and double checked with the existing corresponding report (revised by a cardiologist with over 15 years of experience in cardiac MRI). Infarcted segments were considered transmural if > 50% of the wall thickness was involved. Scars with less than 50% of the wall thickness were classified as subendocardial. No segments with LGE were found in the control group. The average amount of infarcted segments per patient was 3.4 (range: 2–7). Mean scar burden of the patient group was 21.4% ± 3 (range 3–56%). The most frequently infarcted segments were segment 4, 7 and 10, in descending order.

**Table 1:** Demographic characteristics of patients and controls.

	Patients	Controls	p-values	Patients EF > 50%	Patients EF < 50%	p-values
	(n = 46)	(n = 24)		(n = 19)	(n = 27)	
**Patient demographics**						
Sex (female)	41 (5)	22 (2)		18 (1)	23 (4)	
Age (years)	52 ± 19 [39–72]	47 ± 13 [42–67]	0.7	52 ± 7 [39–67]	56 ± 12 [49–72]	0.8
Height (m)	1.69 ± 12 [1.68–1.94]	1.65 ± 15 [1.57–1.9]	0.3	1.69 ± 9 [1.68–1.91]	1.68 ± 14 [1.68–1.94]	0.7
Weight (kg)	79.8 ± 15 [68–103]	76.4 ± 17 [68–94]	0.7	77.2 ± 11 [68–90]	80.6 ± 11 [70–103]	0.6
BMI	27 ± 6 [24–31]	26 ± 3 [24–30]	0.6	27 ± 4 [24–29]	27 ± 5 [24–31]	0.7
**Left ventricular morphology**						
LVEDV (ml,117–200)	178 ± 34 [114–288]	166 ± 37 [81–215]	0.1	172 ± 19 [114–211]	184 ± 27 [145–288]	0.2
LVESV (ml, 31–76)	81 ± 41 [38–195]	67 ± 24 [31–117]	0.4	81 ± 29 [38–160]	86 ± 26 [49–195]	0.8
LVSV (ml, 77–133)	87 ± 18 [57–115]	90 ± 18 [55–115]	0.5	89 ± 18 [57–115]	72 ± 18 [59–103]	0.5
LVEF (**%,** > 52)	46.9 ± 10 [18–62]	58.6 ± 4 [53–69]	0.8	50.9 ± 4 [50–62]	43.6 ± 1 [18–49]	0.8
LV Mass (g, 51–87)	48 ± 17 [31–89]	53 ± 8 [37–99]	0.6	48 ± 17 [31–89]	53 ± 8 [37–99]	0.6
**Global strain**						
GPCS (%)	− 10.5 ± 3	− 20.6 ± 2	0.02	− 12.3 ± 2	− 8.5 ± 2	0.2
GPLS (%)	− 11.8 ± 3	− 18.2 ± 2	0.04	− 13.6 ± 4	− 10.1 ± 2	0.5
GPRS (%)	27.5 ± 6	39.6 ± 4	0.01	33.4 ± 6	21.8 ± 5	0.2
**Infarcted segments**						
Transmural	102	n.a.	–	42	60	0.6
Subendocardial	45	n.a.	–	19	26	0.7

*BMI* body mass index, *LVEDV* left ventricular end-diastolic volume, *LVESV* left ventricular end-systolic volume, *LVSV* left ventricular stroke volume, *LVEF* left ventricular ejection fraction; values in round brackets are standard, cohort specific LV values; values in square brackets represent the value range; patients were further subdivided in two groups (grey labeling): left ventricular EF > 50% (n = 19) and EF < 50% (n = 27).

#### Feature tracking analysis

Global (global peak circumferential [GPCS], global peak longitudinal [GPLS] and global peak radial strain [GPRS]) and segmental (segmental peak circumferential [SPCS], segmental peak longitudinal [SPLS], segmental peak radial strain [SPRS]) strain parameters were calculated from standard balanced SSFP cine sequences using commercially available software (Segment CMR, Medviso, Lund, Sweden) in accordance with the American Heart Association’s 16 segment model as previously described^21^. Datasets of all patients and controls were loaded separately into the software through USB-device and image registration could be started by specific strain icons (one for the short axis stack, a second one for 2-,3-,4-chamber long axis). After image registration, endocardium and epicardium of every slice of the short axis stack (needed for global and segmental circumferential and radial strain) and of 2-,3-,4-chamber long axis (needed for global and segmental longitudinal strain) were manually contoured in end-diastole and in end-systole. Myocardial borders were automatically propagated throughout the cardiac cycle calculating myocardial strain. Contours could be manually corrected throughout the cardiac cycle, if necessary. Mean time duration of data loading, image registration, myocardial contouring until FT results (short axis and 2-,3-,4-chamber long axis) was 10 min 32 s ± 51 s (range 9 min 11 s–12 min 49 s). All FT strain analysis oft the patient group and the control group were perfomed blinded to patient information and LGE images by one reader (reader A: 5 years of experience in cardiac imaging). Twenty-four cases were randomly chosen for performing interobserver agreement (reader B: 1 year of experience in cardiac imaging), blinded to results of the first reader. For further global strain analysis, the patient group was subdivided in patients with preserved and reduced LVEF (Table 1).

#### Localization of potentially infarcted segments in circumferential strain calculations and in cine images

**Figure 1:**
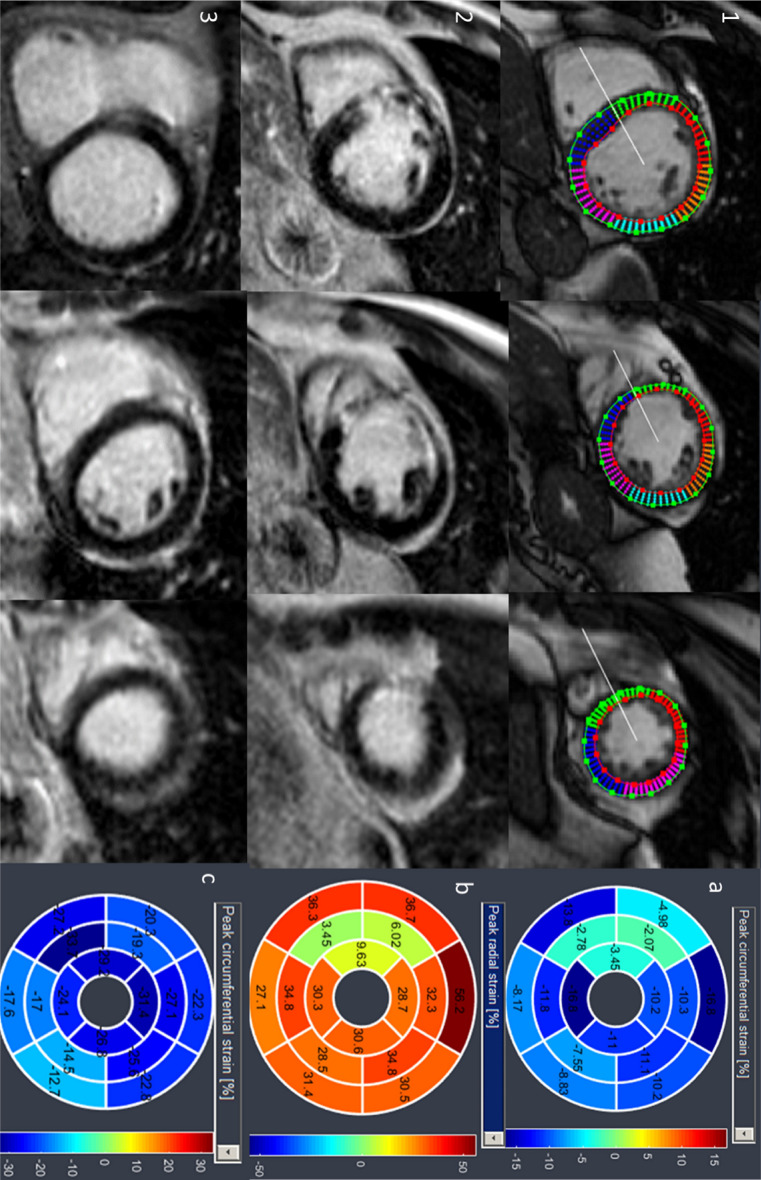
Basal, midventricular and apical section of cine short axis with tracked myocardial borders for segmental circumferential strain calculation (1) and LGE (2) in the same patient with RIVA infarction (segment 2, 8, 9, 15). Based on the deformation of tracked myocardial borders, polar plot maps of calculated segmental circumferential strain (**a**) and segmental radial strain (**b**) are depicted. Subendocardial infarction in segment 2 shows no segmental radial strain impairement. Basal, midventricular and apical LGE sections and circumferential strain values of a healthy control (3, **c**).

Reader A and B were advised to localize potentially infarcted segments in segmental circumferential strain calculations (endo-/epicardially contoured short axis cine images and resulting polar plot strain map, Fig. 1) as well in the corresponding cine short axis images by detecting wall motion abnormalities with visual wall motion assessment. In both methods, all 16 segments from a basal, midventricular and apical section were evaluated through a cardiac cycle and segments were classified in a binary manner (infarcted or not infarcted).

Both methods (performed for all patients and controls) were presented to the readers in random order, blinded to patient information and LGE images. The readers perfomed this evaluation separatly, blinded to the results of each other.

### Statistical analyses

Statistical analyses were performed using commercially available software (SPSS, release 20.0; SPSS, Chicago, IL, USA). Quantitative data are expressed as means ± standard deviations and categoric data are expressed as numbers or percentages. The Kolmogorov–Smirnov Test was used to evaluate normal distribution. Depending on distribution of normality, two-tailed paired *t*-tests and Wilcoxon signed rank were used to compare global and segmental strain values as well as to compare infarcted segments found in LGE, circumferential strain calculations and by visual wall motion assessment. The Intraclass Correlation Coefficient (ICC) was used to determine interobserver agreement in strain calculations and to determine interobserver agreement in identified infarcted segments in circumferential strain calculations and in visual wall motion assessment. ICC = 0.50–0.75 was considered moderate, ICC = 0.75–0.9 was considered good and ICC > 0.9 was considered excellent agreement^29^. Receiver operating characteristics (ROC) curve analysis was performed to determine the cut-offs of segmental strain values and area under the curve (AUC) for circumferential and radial strain in order to differentiate infarcted from remote myocardium. ROC curve analysis was not performed for segmental longitudinal strain due to lacking significance between strain values in infarcted and remote myocardium. Statistical significance was assumed at a p-value below 0.05.

## Results

### Left ventricular ejection fraction (LVEF) and global strain

**Figure 2:**
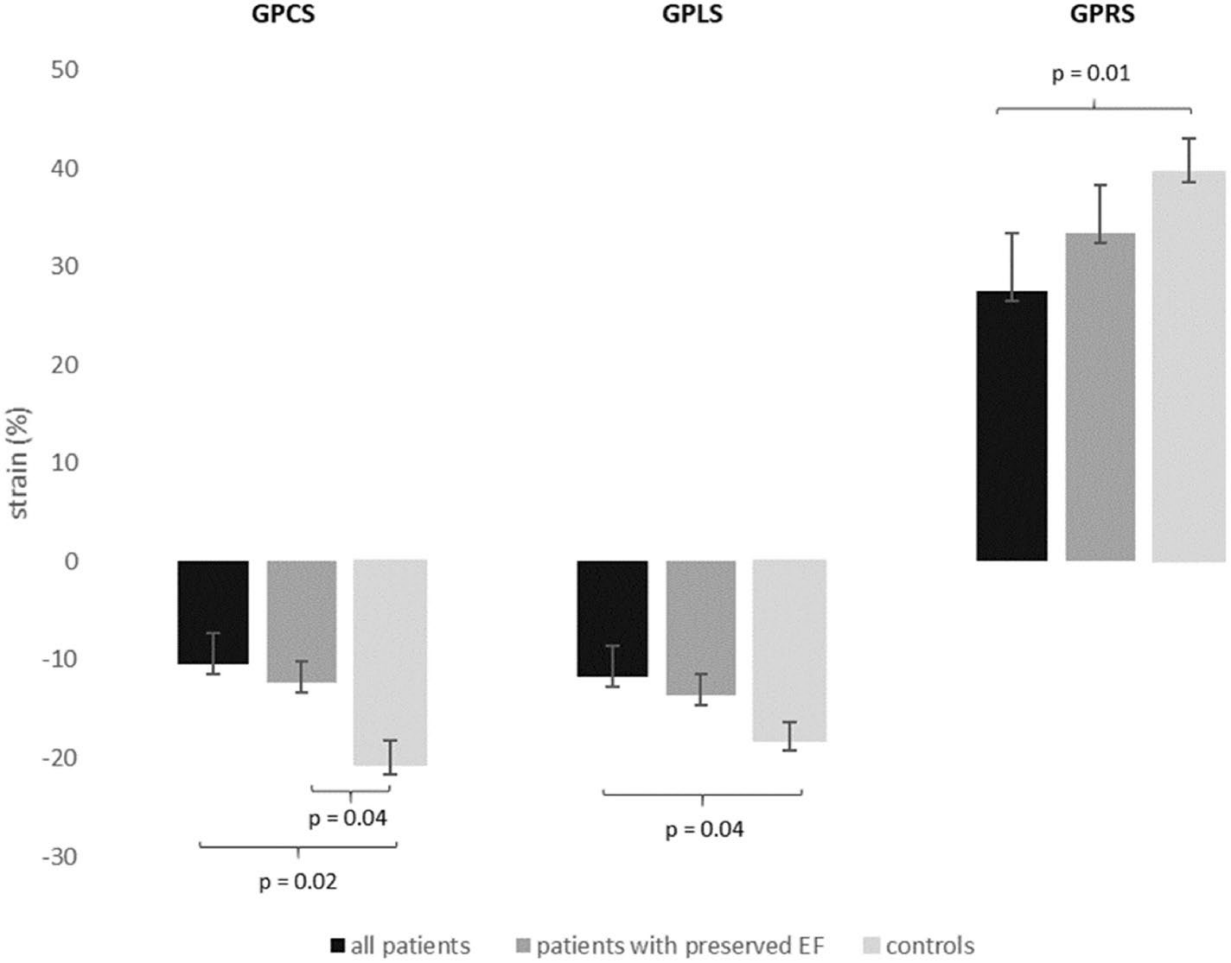
Global strain values in all patients, patients with preserved EF (LVEF > 50%) and healthy controls. Brackets signalize significantly different strain values between groups. *(LV)EF* (left ventricular) ejection fraction, *GPCS* global peak circumferential strain, *GPLS* global peak longitudinal strain, *GPRS* global peak radial strain.

Overall LVEF was 46.9 ± 10% in the patient group and 58.6 ± 4% in the control group (p = 0.8, Table 1). Global strain values were reduced in patients compared to healthy controls (GPCS − 10.5% ± 3 vs. − 20.6% ± 2, p = 0.02; GPLS – 11.8% ± 3 vs. – 18.2% ± 2, p = 0.04; GPRS 27.5% ± 6 vs. 39.6% ± 4; p = 0.01, Table 1), interobserver agreement was good or excellent (Table 2). Also patients with preserved LVEF (LVEF > 50%, 19 patients)^30^ had markedly reduced GPCS compared to healthy individuals (− 12.3% ± 2 vs. − 20.6% ± 2, p = 0.04), while GPLS and GPRS was not significantly impaired (GPLS − 13.6% ± 4 vs. − 18.2% ± 2, p = 0.2; GPRS 33.4% ± 6 vs. 39.6% ± 5, p = 0.3) (Fig. 2). Remote myocardium in infarct patients showed reduced peak circumferential strain compared to controls (12.9% ± 3 vs. − 20.6% ± 2, p = 0.04), peak longitudinal strain (− 12.8% ± 3 vs. − 18.2% ± 2, p = 0.2) and peak radial strain (31.4% ± 5 vs. 39.6% ± 5, p = 0.3) on the other hand was only mildly impaired in remote myocardium of infarct patients compared to healthy individuals.

**Table 2:** Interobserver agreement.

	ICC
**Global strain**	
GPCS	0.913 [95% CI:0.862–0.922]
GPLS	0.841 [95% CI:0.804–0.878]
GPRS	0.901 [95% CI:0.861–0.931]
**Segmental strain**	
SPCS	0.899 [95% CI:0.862–0.922]
SPLS	0.722 [95% CI:0.701–0.747]
SPRS	0.814 [95% CI:0.797–0.902]

*GPCS/SPCS* global/segmental peak circumferential strain, *GPLS/SPLS* global/segmental peak longitudinal strain, *GPRS/SPRS* global/segmental peak radial strain.

### Segmental strain

From 736 segments 147 segments were diagnosed with scars on LGE images (20%), of which 102 were considered transmurally infarcted and 45 were subendocardially infarcted.

#### Segmental circumferential strain

**Figure 3:**
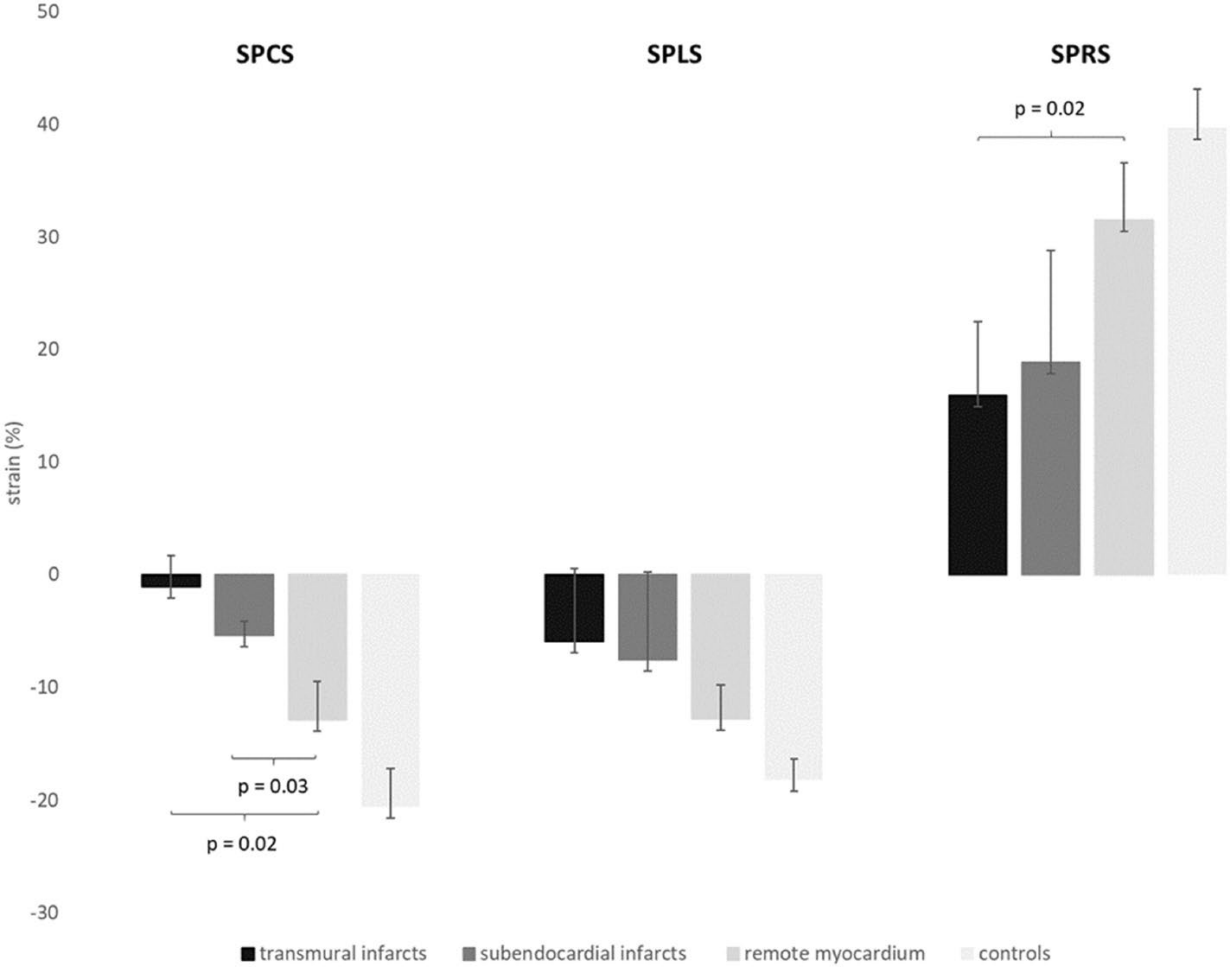
Segmental strain values in patients and healthy controls. Brackets signalize significantly different strain values between groups. Significance between infarcted segments and controls are presumed and thus not marked. *SPCS* segmental peak circumferential strain, *SPLS* segmental peak longitudinal strain, *SPRS* segmental peak radial strain.

**Figure 4:**
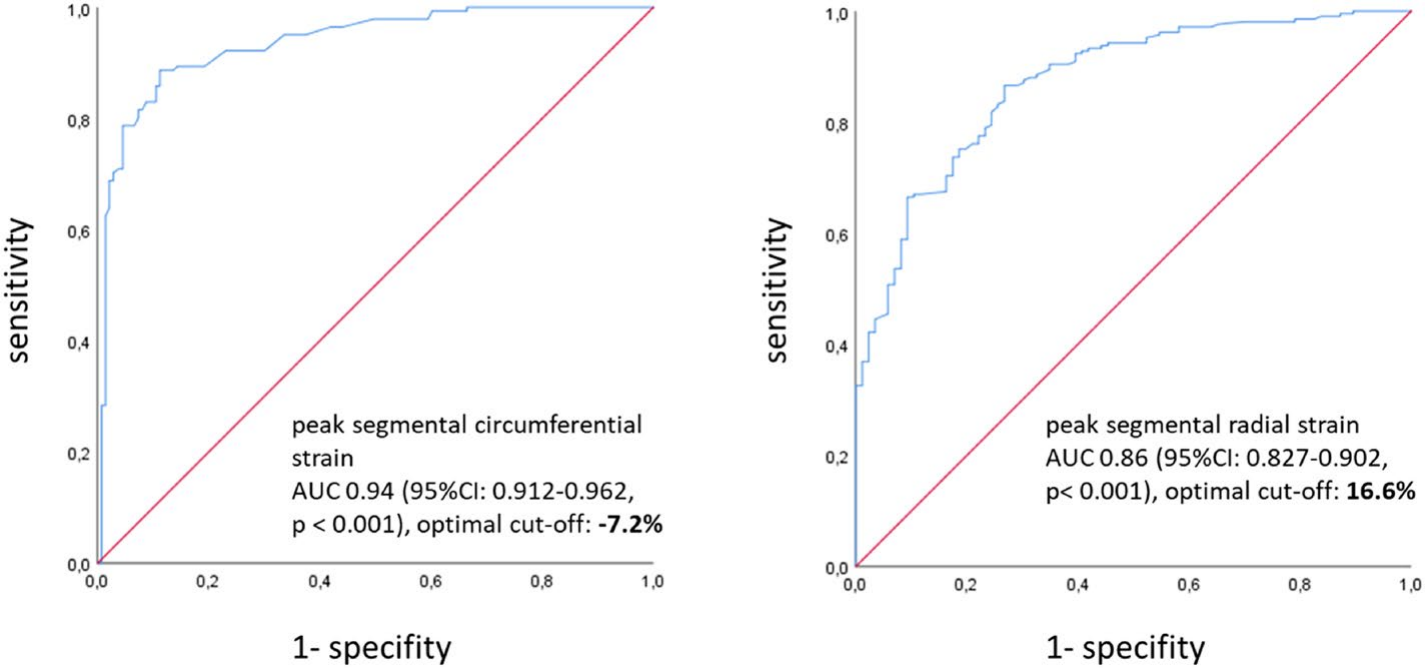
ROC curves for distinguishing infarcted and remote myocardium based on strain parameters. In SPCS the optimal cut-off is − 7.2% (sensitivity of 89.4% and specificity of 85.7%) and in SPRS the optimal cut-off is 16.6% (sensitivity of 83.3% sensitivity and specificity of 76.5%). *ROC* Receiver operating characteristic, *SPCS* segmental peak circumferential strain, *SPRS* segmental peak radial strain.

Among the patient population mean segmental peak circumferential strain (SPCS) was significantly impaired in subendocardial infarcts (− 5.4% ± 2) and even more in transmurally infarcted segments (−  1.2% ± 3) compared to mean SPCS of remote myocardium (− 12.9% ± 3, p = 0.02 and 0.03) (Fig. 3) with good interobserver agreement (Table 2). In ROC analysis the optimal cut-off value for SPCS for discriminating scar tissue from remote myocardium was − 7.2% with a sensitivity of 89.4% and specificity of 85.7%, AUC 0.94 [0.912–0.962] (Fig. 4, left image).

#### Segmental longitudinal strain

Among the patient population mean segmental peak longitudinal strain (SPLS) was mildly impaired in subendocardially (− 7.5% ± 8) and transmurally (− 5.9% ± 7) infarcted segments compared to mean SPLS of remote myocardium (− 12.8% ± 3, p = 0.3 and 0.4) (Fig. 3). Interobserver agreement was moderate (Table 2).

#### Segmental radial strain

Mean segmental peak radial strain (SPRS) was mildly impaired in subendocardially infarcted segments in the patient cohort (18.9% ± 10), but significantly impeded in transmurally infarcted segments (15.9% ± 6) compared to SPRS of remote myocardium (31.4% ± 5; p = 0.3 and 0.02) (Fig. 3), interobserver agreement was good (Table 2). The optimal cut-off value for SPRS for discriminating scar tissue from remote myocardium was 16.6% with a sensitivity of 83.3% and specificity of 76.5%, AUC 0.86 [0.827–0.902] (Fig. 4, right image).

#### Localization of infarcted segments in circumferential strain calculations and by visual wall motion assessment

**Figure 5:**
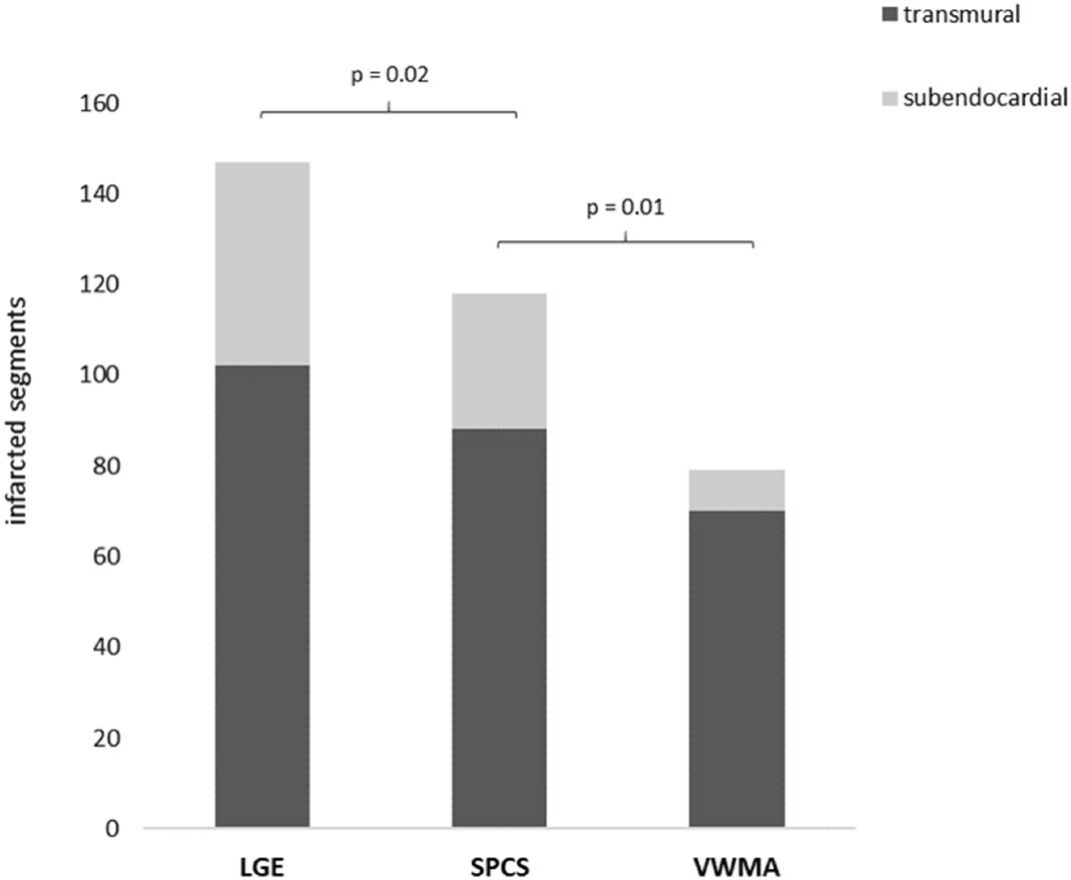
Localization of infarcted segments in segmental circumferential strain calculations (SPCS) showed significantly more infarcted segments (80.3%) than visual assessment of wall motion abnormalities in cine images (53.7%); infarcted segments in LGE images served as gold standard. Brackets signalize significance between groups. *LGE* late gadolinium enhancement, *SPCS* segmental peak circumferential strain, *VWMA* visual wall motion assessment.

Localization of potentially infarcted segments based on segmental circumferential strain calculations (endo-/epicardially contoured short axis cine images and resulting polar plot strain map, Fig. 1) revealed 118 infarcted segments from 147 infarcted segments (80.3%, 88 transmural, 30 subendocardial; Fig. 5) with excellent interobserver agreement (ICC 0.904, 95%CI 0.845–0.933). 29 infarcted segments were not detected (24 subendocardial and 5 transmural), among them one patient with only a small transmural scar in segment 15. All other patients diagnosed with scars in LGE images had at least one impaired segment in circumferential strain calculations and the missed infarcted segments were localized adjacent to already diagnosed infarcts.

In visual wall motion assessment of cine short axis images 79 segments from 147 infarcted segments had wall motion abnormalities and were classified as “infarcted” (53.7%, 70 transmural, 9 subendocardial; ICC 0.811, 95%CI: 0.782–0.859), especially infarcts in the posterior wall (segments 4, 10, 15) were missed (41 from 68 missed infarcts, 60.3%). Both readers did not detect any wall motion abnormalities in the control group or in remote segments of the patient cohort.

## Discussion

This study examined global and segmental myocardial deformation indices in patients with chronic ischemic scars and the feasibility of using segmental strain for detection of chronic infarcts in non- contrast cine images.

Main results of this study are: (a) global strain values, especially GPCS, are markedly impaired in patients with infarcts, also in patients with preserved EF compared to controls (b) both transmurally and subendocardially infarcted segments show significantly reduced segmental circumferential strain compared to remote myocardium in infarct patients (c) 80% of infarcted segments could be correctly localized from segmental circumferential strain calculations based on non-contrast cine images, while only 54% of infarcted segments could be detected by visual wall motion assessment of cine images. After myocardial infarction, scar tissue replacing myofibroblasts leads to altered global and segmental strain behavior of the heart. Recent studies focused on the investigation of global strain parameters in patients with acute and chronic infarcts, revealing reduced global longitudinal and global circumferential strain in these patients^23,24^. In the last decade, studies that analyzed segmental strain in patients with infarction show heterogenous results, in particular reduced accuracy and reproducibility of segmental strain values was reported^25,26^. Those studies used optical flow- based FT methods with tracking of myocardial boundaries. The FT software used in this study is based on a non-rigid algorithm for image registration with tracking of the entire image content with higher reliability and interobserver agreement for segmental strain values in previous studies^21,22^.

In line with current study results, GPLS, GPCS and GPRS were impaired in our patient cohort compared to healthy controls^31^. Strain parameters were able to detect subclinical impairment of cardiac function in infarct patients with normal ejection fraction, concluding that strain is a more sensitive parameter for cardiac function compared to LVEF^32^. Moreover, we discovered that remote myocardium in infarct patients has lower mean strain values compared to healthy controls, suggesting subclinical changes in strain behavior of remote myocardium of patients after ischemia and scar formation^33,34^.

Both transmural and subendocardial scars showed significantly impaired mean SPCS compared to remote myocardium as well as impeded mean SPRS in transmurally infarcted segments. There are no definite cut-off values published for discriminating infarcts from remote myocardium. In our patient cohort the derived cut-off value was − 7.2% for segmental circumferential strain (below which segments are considered remote) and 16.6% for radial stain (above which segments are considered remote). Cut-off values for circumferential strain were comparable with those from other research groups^26,28^, but the cut-off value for radial strain was higher than in other studies, mostly due to already higher normal value for radial strain in our patient group. In our patient group, mean segmental longitudinal strain was not significantly impaired in scar tissue compared to remote myocardium.

Based on the observation how segmental circumferential strain is affected in chronic infarcts, we examined infarct localization in segmental circumferential strain calculations (endo-/epicardially contoured short axis cine images and resulting polar plot strain map) for all patients and controls by two readers, blinded to LGE images and clinical information. While visual wall motion assessment based on non-contrast cine short axis images detected about half of all infarcted segments (53.7%), 80% of infarcted segments could be localized correctly in circumferential strain calculations. One patient with a small transmural scar in the posterior wall was not detected in segmental circumferential strain calculation. Further analysis of the other 28 missed infarcted segments showed that those segments were localized adjacent to already as “infarcted” classified segments and were mostly subendocardial. Those observations emphasize that segmental strain clearly improves the amount of identified old scars over the visual assessment of cine images.

This method seems to be promising for scar detection in patients who cannot receive or refuse gadolinium or patients in reduced condition that abort the scan before contrast application. Some artificial intelligence-based techniques have tried to detect scar tissue in non-contrast cine MRI sequences^8,35^, but the underlying studies are often in a proof-of-concept stage, require more extensive system integration or are not yet practicable in the clinical setting. FT is already in clinical use and can be easily incorporated in the clinical routine.

Since local T1 mapping in detection chronic infarcts scar detection, combined use of segmental strain calculations and T1 mapping could probably enhance the diagnostic accuracy of scar localization in non-contrast MRI protocols even more^36^.

## Limitations

Some study limitations must be acknowledged. This is a retrospective analysis of data from 46 patients and 24 age- and gender matched controls and most patients and controls are male. Equal amount of both genders should be investigated, since it has been shown, that global strain values differ between men and women^37,38^. Further studies with more patients are needed to establish reliable cut-off segmental strain values for remote and scarred myocardium. The benefit of segmental circumferential strain calculations over visual wall motion evaluation based on cine images should be investigated in a prospective setting. Even though, no segments with wall motion abnormalities and lack of LGE were detected in our small study cohort, “stunned” (in acute ischemia) or “hibernated” (in chronic ischemic conditions) myocardial segments^39^ might occur in a prospective patient cohort with patients with unstable CAD or patients with recent myocardial infarction and may provide false positive results in strain calculations.

## Conclusion

Global strain parameters are impaired in patients with chronic infarcts compared to healthy individuals. In our feasibility study, 80% of infarcted segments could be detected in segmental circumferential strain calculations based on non-contrast cine images, while visual wall motion assessment of cine images estimated only about 54% of all infarcted segments. Although some questions remain unanswered, in particular the influence of not infarcted myocardium with wall motion abnormalities (“stunned” or “hibernated” myocardium) on segmental strain, this method might be helpful for detection of chronic scars in patients with contraindications for gadolinium, patients who decline contrast or when LGE images are non-diagnostic. Further prospective studies are needed, but this technique may be one small step in reducing gadolinium in cardiac MRI protocols in the future.

## Abbreviations

AUC: Area under the curve
FT: Feature tracking
GPCS: Global peak circumferential strain
GPLS: Global peak longitudinal strain
GPRS: Global peak radial strain
ICC: Intraclass correlation coefficient
i.v.: Intravenous
LGE: Late gadolinium enhancement
LV: Left ventricle/left-ventricular
LVEDV: Left ventricular end-diastolic volume
LVEF: Left ventricular ejection fraction
LVESV: Left ventricular end-systolic volume
LVSV: Left ventricular stroke volume
MRI: Magnetic resonance imaging
MI: Myocardial infarction
ms: Milliseconds
min: Minute(s)
ROC: Receiver operating characteristics
s: Seconds
SPCS: Segmental peak circumferential strain
SPLS: Segmental peak longitudinal strain
SPRS: Segmental peak radial strain
SSFP: Steady-state free precession
T: Tesla
